# Size-selective mortality fosters ontogenetic changes in collective risk-taking behaviour in zebrafish, *Danio rerio*

**DOI:** 10.1007/s00442-022-05256-y

**Published:** 2022-10-01

**Authors:** Tamal Roy, Robert Arlinghaus

**Affiliations:** 1 Department of Fish Biology, Fisheries and Aquaculture, Leibniz Institute of Freshwater Ecology and Inland Fisheries, Müggelseedamm 310, 12587 Berlin, Germany; 2 Division of Integrative Fisheries Management, Department of Crop and Animal Sciences, Faculty of Life Sciences, Humboldt-Universität zu Berlin, Philippstrasse 13, Haus 7, 10115 Berlin, Germany

**Keywords:** Collective boldness, Size-selective harvesting, Fisheries-induced evolution, Behavioural plasticity, Zebrafish

## Abstract

Size-selective mortality is common in fish populations and can operate either in a positive size-selective fashion by harvesting larger-than-average fish or be negatively size-selective by harvesting smaller-than-average fish. Through various mechanisms (like genetic correlations among behaviour and life-history traits or direct selection on behaviour co-varying with growth rate or size-at-maturation), size-selection can result in evolutionary changes in behavioural traits. Theory suggests that both positive and negative size-selection without additional selection on behaviour favours boldness, while evolution of shyness is possible if the largest fish are harvested. Here we examined the impact of size-selective mortality on collective boldness across ontogeny using three experimental lines of zebrafish (*Danio* *rerio*) generated through positive (large-harvested), negative (small-harvested) and random (control line) size-selective mortality for five generations and then relaxed selection for 10 generations to examine evolutionarily fixed outcomes. We measured collective risk-taking during feeding (boldness) under simulated aerial predation threat, and across four contexts in presence/absence of a cichlid. Boldness decreased across ontogeny under aerial predation threat, and the small-harvested line was consistently bolder than controls. The large and small-harvested lines showed higher behavioural plasticity as larvae and developed personality earlier compared to the controls. The large-harvested line showed increased variability and plasticity in boldness throughout ontogeny. In the presence of a live predator, fish did not differ in boldness in three contexts compared to the controls, but the large-harvested line showed reduced behavioural plasticity across contexts than controls. Our results confirmed theory by demonstrating that size-selective harvesting evolutionarily alters collective boldness and its variability and plasticity.

**Supplementary Information:**

The online version contains supplementary material available at 10.1007/s00442-022-05256-y.

## Introduction

Intensive fishing as well as natural mortality is typically size-selective in nature, meaning that fish of certain size-classes are preferentially harvested from populations (Hamilton et al. 2007; Heino et al. 2015; Jørgensen and Holt 2013). Most fishing gears selectively capture the large and fast-growing fish in populations (positive size-selection) (Law 2007), while most natural predators target smaller-than-average sizes (negative size-selection) (Edeline and Loeuille 2021; Stige et al. 2019). Elevated mortality, even if unselective for size, is known to generally select for a fast life-history characterized by rapid maturation and fast juvenile growth, elevated reproductive investment and reduced adult growth and longevity (Hamilton et al. 2007; Heino et al. 2015; Jørgensen and Holt 2013; Laugen et al. 2014; Uusi‐Heikkilä et al. 2015; Wootton et al. 2021). Positive size-selection reinforces such life-history adaptations and adds pressures to mature earlier and at smaller sizes at the expense of post-maturation growth rate (Andersen et al. 2018; Jørgensen et al. 2007). While empirical evidence exists for the evolution of a fast life-history due to intensive and positive size-selective mortality (Jørgensen et al. 2007; Uusi‐Heikkilä et al. 2015; van Wijk et al. 2013), the evolutionary direction in which personality traits like boldness change in response to either elevated and unselective, or elevated and size- or otherwise trait-selective harvesting has so far been studied only in a few theoretical models (Andersen et al. 2018; Claireaux et al. 2018; Jørgensen and Holt 2013). Here, we investigate the impact of size-selective mortality on boldness as a collective personality trait using experimental evolution in zebrafish (*Danio rerio*).

The pace-of-life hypothesis suggests that adaptations towards a fast life-history should be correlated with an increased boldness (Biro and Stamps 2008; Réale et al. 2010) in order to accumulate resources for rapid growth and faster maturation (Jørgensen and Holt 2013; Montiglio et al. 2018). Population models that integrate behavioural processes involved in growth-mortality trade-offs have suggested that unselective (Claireaux et al. 2018; Jørgensen and Holt 2013) and size-selective harvesting would bring about increased boldness in exploited fish across a large gradient of size-selectivity (Andersen et al. 2018). However, Andersen et al. (2018) predicted that if size-selection is directed towards adult fish much larger than size-at-maturation, then evolution of shy behavioural phenotypes even in purely size-selected fisheries without any additional direct selection on behaviour could be possible. Because most fishing gears are not just selective of body-size but also selective of behavioural traits like boldness, behaviour-selective harvesting can additionally favour shy behavioural phenotypes (timidity syndrome: Arlinghaus et al. 2017). While theory predicts that the evolution of both elevated boldness or increased shyness is possible in association with the evolution of fast life-history (Andersen et al. 2018), there is no experimental evidence for these outcomes in scenarios of different types of size-selection. Laboratory (Polverino et al. 2018) and field studies (Dhellemmes et al. 2021) have shown that a positive association between boldness and fast life-history based on the pace-of-life hypothesis (Réale et al. 2010) might break if populations are exposed to high adult mortality or other ecological gradients (Laskowski et al. 2021; Royauté et al. 2018). Thus, a fast life-history may be associated with reduced boldness in fish populations adapted to positive size-selective harvesting.

A fast life-history evolves to cope with high adult mortality thereby allowing fish to reproduce early in life. Fish genetically predisposed to exert a fast-life history are expected to be bolder as juveniles because they must acquire the resources necessary for development of gonads early in life and therefore must take risks during foraging (Claireaux et al. 2018; Jørgensen and Holt 2013). But most predators are gape-limited and natural mortality due to predation is higher for juveniles than adults (Gislason et al. 2010; Lorenzen 2000). Thus, fish genetically predisposed to a fast life-history could be more risk-averse (i.e. shyer) in the juvenile stage and be bolder in the adult stage when they have a reached a size that renders them less vulnerable to predation (Ballew et al. 2017). However, fish with a fast life-history tend to remain smaller post-maturation as a trade-off with elevated reproductive investment compared to the fish with slow life-history (Dunlop et al. 2009; Uusi‐Heikkilä et al. 2015; Wootton et al. 2022). Because body-size is negatively correlated with natural predation (Lorenzen 2006), it is likely that life-history transition changes boldness expression of the “faster” fish post maturation resulting in lower boldness relative to the fish demonstrating a slow life-history in the adult stage. Thus, boldness expression may change through development in an adaptive fashion commensurate with life-history adaptations to different size-selection pressures. Indeed, animal personality expression changes with maturation, and tends to be stable within, but not across life stages (Cabrera et al. 2021; Groothuis and Trillmich 2011). Studies across different fish species have mostly revealed changes in individual personality (i.e. consistent individual differences in behaviour across time and contexts: Réale et al. 2007), and behavioural variability (between-individual variation in behaviour: Dingemanse and Dochtermann 2013) and plasticity (within-individual variation in behaviour: Stamps et al. 2012) across developmental stages. For instance in mosquitofish *Gambusia holbrooki*, no evidence of personality was found in juveniles because of high behavioural plasticity but personality emerged in the subadult stage when plasticity decreased, though behavioural variability did not change across ontogenetic age (Polverino et al. 2016b). Consistent differences in individual behaviour (Jolles et al. 2017) and the attraction and interaction rules among social individuals (Couzin and Krause 2003; Hinz and de Polavieja 2017) drive group behaviour. Hence, changes in behavioural variability, plasticity and personality of individuals through development may result in changes in group or collective personality (Bengston and Jandt 2014). Moreover, collective personality can change post maturation because the priorities of individuals and groups change from growth and survival in the juvenile stage to mainly survival and reproduction after maturation (Bengston and Jandt 2014).

Group personalities in fish are known to change over different time scales. For example, sticklebacks *Gasterosteus aculeatus* showed consistent variation in collective motion traits within trials and between days (MacGregor and Ioannou 2021), and consistent group structure and movement dynamics across different contexts (Jolles et al. 2018). But there is less evidence of change in variability, plasticity, and personality among groups across developmental stages. Studying groups rather than individual behaviour is more ecologically relevant in gregarious fish species where living in groups offers adaptive advantages (Krause et al. 2002). Many exploited fish, especially the small pelagic species, are group-living in nature (Croft et al. 2003) but whether intensive size-selection alters ontogenetic trajectories of group personality is currently unknown. Because size-selection alters individual personality traits (Biro and Post 2008; Diaz Pauli et al. 2019; Sbragaglia et al. 2019a) and may erode behavioural variability (Arlinghaus et al. 2017; Monk et al. 2021), alteration of individual plasticity, and thereby personality of groups, is possible in response to trait-selective fisheries (Guerra et al. 2020; Louison et al. 2018). Recent studies experimentally tested the impact of size-selection on group behaviour in adult zebrafish, a gregarious species (Suriyampola et al. 2016), at two time points and found that positive size-selection resulted in increased shoaling while negative size-selection resulted in decreased shoaling but increased group risk-taking behaviour (Sbragaglia et al. 2021, 2022). We built on this work to understand if size-selective mortality affects collective risk-taking behaviour across ontogeny.

We used three experimental lines of zebrafish generated through positive (large-harvested), negative (small-harvested) and random (control) size-selective mortality for five successive generations followed by no selection for 10 generations to investigate the impact of size-selection on group boldness through ontogeny. The large-harvested line mimics the common scenario in global fisheries where large-sized individuals are predominantly harvested, while the small-harvested line resembles fisheries where maximum-size limits exist or in case of natural predation where mainly the smallest size classes are eaten. From F_11,_ the experimental lines differed in life-history (Uusi‐Heikkilä et al. 2015) and behavioural traits (Roy et al. 2021; Sbragaglia et al. 2019a, 2021, 2022; Uusi‐Heikkilä et al. 2015), and these differences were accompanied by changes in broad-scale gene expression and allele frequencies (Sbragaglia et al. 2021; Uusi‐Heikkilä et al. 2015, 2017). Thus, the phenotypic differences have genetic underpinnings and are not just the result of phenotypic plasticity. Behavioural studies revealed that in the small-harvested line, juveniles (30 day old) and adult females were bolder and more explorative in an open field test when tested individually (Sbragaglia et al. 2019a; Uusi‐Heikkilä et al. 2015), while groups of adults (230 and 240 day old) were bolder while feeding under simulated predation threat (Sbragaglia et al. 2020) than the control lines. The large-harvested line fish did not differ in boldness from the control line as individuals (Sbragaglia et al. 2019a; Uusi‐Heikkilä et al. 2015) or groups (Sbragaglia et al. 2021). These studies focused on one developmental stage, and on individual, rather than group behavioural phenotypes. Here we measured group risk-taking behaviour (boldness) in the selection lines in two experiments across development and different predation risk contexts to get a complete picture of the evolution of boldness in response to size-selection.

We first investigated how collective risk-taking to feed in presence of a simulated aerial predator (Sbragaglia et al. 2020; Ward et al. 2004) changed from larval to adult stages among the selection lines. Because fish may respond differently to aerial and aquatic predators owing to conflicting selection pressures exerted by them (Godin and Clark 1997; Templeton and Shriner 2004; Wund et al. 2015), we also tested boldness in presence of different cues (visual and/or olfactory) from a live predatory fish, the convict cichlid (*Amatitlania nirgrofasciata*) (Sailer et al. 2012; Toms and Echevarria 2014). We expected that boldness will emerge as a collective personality trait through ontogeny among all selection lines, similar to what is known from studies on individual personality traits in fish (Edenbrow and Croft 2011; Polverino et al. 2016b). Given that the large and small-harvested zebrafish lines are of fast and slow life-histories (Uusi‐Heikkilä et al. 2015), and considering that Andersen et al. (2018) theoretically predicted that size-selection tends to generate bold fish unless only the largest fish are selected, we hypothesized that both selection lines would show elevated collective boldness compared to the controls, and this effect would be more pronounced in the small-harvested line. We also expected that maturation would change boldness, with fish groups of the large-harvested line showing no difference in behaviour compared to the controls in juvenile stage (as juvenile growth rate of large-harvested and control lines were similar; Uusi‐Heikkilä et al. 2015) while being generally bolder as adults but less bold than the control line after maturation to adjust their behaviour to the now smaller body-size. We further expected that similar to ontogenetic responses in individuals in other fish species (Polverino et al. 2016b), plasticity in boldness would decrease while variability would remain unchanged across ontogeny among all selection lines. Disruptive selection due to harvesting may alter trait variability (Landi et al. 2015; Monk et al. 2021). For example in pike (*Esox lucius*), fishing mortality increases variability in somatic growth rate and size-at-age (Edeline et al. 2009), and in zebrafish, large size-selection leads to increased variation in body size (Uusi-Heikkilä et al. 2016). Here we expected that behavioural variability and plasticity at different stages of development and contexts would be higher in the large-harvested line. This is because of their internal conflict between reaping resources through foraging and avoiding being predated upon due to their smaller body-size, and increased vigilance that may allow them to tune their behaviour with different degrees of predation (Sbragaglia et al. 2022). By contrast, we expected the small-harvested line to be consistently bolder than controls and show less behavioural variability and plasticity through ontogeny and across different contexts as this line is generally bold (Sbragaglia et al. 2021), larger in size compared to controls and more responsive to social cues (Sbragaglia et al. 2022).

## Material and methods

### Selection lines

We used F_16_ of the selection lines of zebrafish (large-, random- and small-harvested lines, each with a replicate, i.e. six lines in total), described in Uusi‐Heikkilä et al. (2015). These lines were produced by subjecting a wild-population of zebrafish to intensive selection (i.e. 75% harvest rate per generation) for five successive generations (F_1_ to F_6_), and thereafter stopping selection for the subsequent 10 generations to remove maternal effects (Moore et al. 2019) and breeding them randomly. 25% of the smallest and largest individuals were used as parents in successive generations in the large- and small-harvested line. Simultaneously, 25% of random individuals were selected for reproduction every generation to produce the control group. Fish were harvested in the subsequent generations based on when 50% of the control line fish became mature. The selection lines were assessed for differences in body-size and key life-history traits from F_11_ onwards and the large-harvested line evolved a fast life-history characterized by elevated reproductive investment, smaller terminal body-size, early maturation and reduced adult growth (Uusi‐Heikkilä et al. 2015). The small-harvested line evolved a slow life-history characterized by reduced reproductive investment and no change in adult body-size compared to the control line (Uusi‐Heikkilä et al. 2015). Among-generation assays of growth trajectory using Lester biphasic growth models conducted at F_9_, F_11_ and F_13_ demonstrated that the selection lines maintained the evolved differences in body-size and growth rate (Roy et al. 2021; Sbragaglia et al. 2019b, 2021). Here we report differences in body-size in F_16_ (ESM, Fig. S1). We assumed that differences in key life-history traits among the selection lines were maintained, and we measured evolutionary fixed outcomes of size-selection on behaviour.

We housed the F_15_ fish of selection lines in laboratory in six round holding tanks (diameter: 79 cm, height: 135 cm, volume: 320 l) at a density of approximately 1000 per tank under 12:12 Light: Dark cycle. The water temperature was maintained at 27 °C by a circulation system, and fish were fed twice daily with commercial flake food (TetraMin Tropical). For producing the F_16_ fish, we stocked six 5-L spawning boxes (total 36) each with four males and two females (Roy et al. 2021; Uusi‐Heikkilä et al. 2010) selected randomly from the F_15_ population, and allowed them to breed. We pooled the embryos produced from each line and transferred 80 embryos into 10 3-L boxes (eight per box). We used 480 fish (i.e. 8 fish × 60 groups; 10 groups per replicate line, 20 groups per treatment) in total and tracked the behaviour of fish through ontogeny. The fish were fed twice a day with powdered flake food in the larval and juvenile stages, and like F_15_ fish when adult.

### Risk-taking under simulated aerial predation

**Fig. 1:**
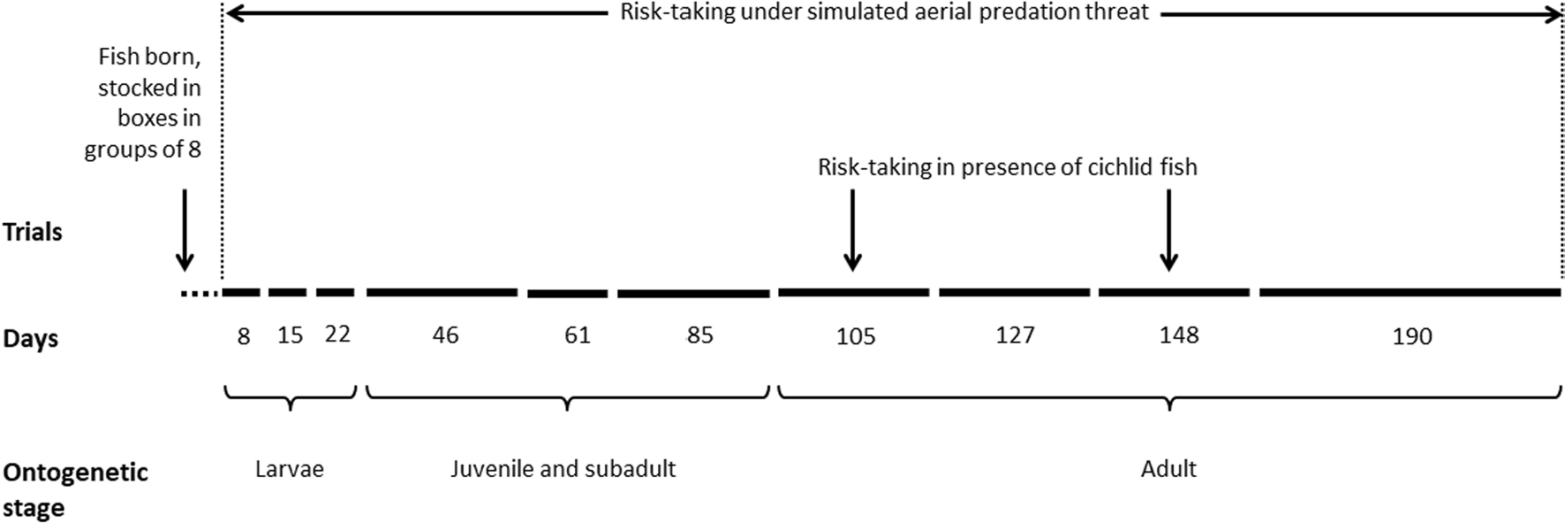
Experimental timeline. Fish across selection lines were tested for risk-taking behaviour in presence of an aerial predation threat from age 8 to 190 dpf, and in presence of a cichlid fish at 90–100 dpf and 132–142 dpf age

We tested risk-taking to feed under simulated aerial predation threat among groups of fish across selection lines using a tank diving paradigm employed previously by Sbragaglia et al. (2021). Collective risk-taking behaviour is a repeatable trait in zebrafish (Sbragaglia et al. 2019b, 2022) and is a group-level personality trait (Bengston and Jandt 2014; Jolles et al. 2018). We tested 60 groups (10 from each replicate line, 20 per treatment) or 480 fish in total. We conducted the assay every week from 8 to 22 days post fertilization or dpf (larval stages), at 45 dpf (juvenile), 61 and 85 dpf (subadult stages), and then every three weeks till 148 dpf and after six weeks at 190 dpf (adult stages) (Alfonso et al. 2020). Behavioural changes in laboratory reared zebrafish is associated with morphological and physiological changes during larval (8–21 dpf), juvenile (21–60 dpf including metamorphosis around 45 dpf), subadult (60–90 dpf) and adult (90–190 dpf) stages (Alfonso et al. 2020; Stednitz and Washbourne 2020). Though the maturation schedules of our size-selected lines are different with both the large-harvested and small-harvested line maturing earlier at smaller sizes than the controls (Uusi‐Heikkilä et al. 2015), we tested them at the abovementioned stages to have an uniform experimental timeline (Fig. 1).

**Fig. 2:**
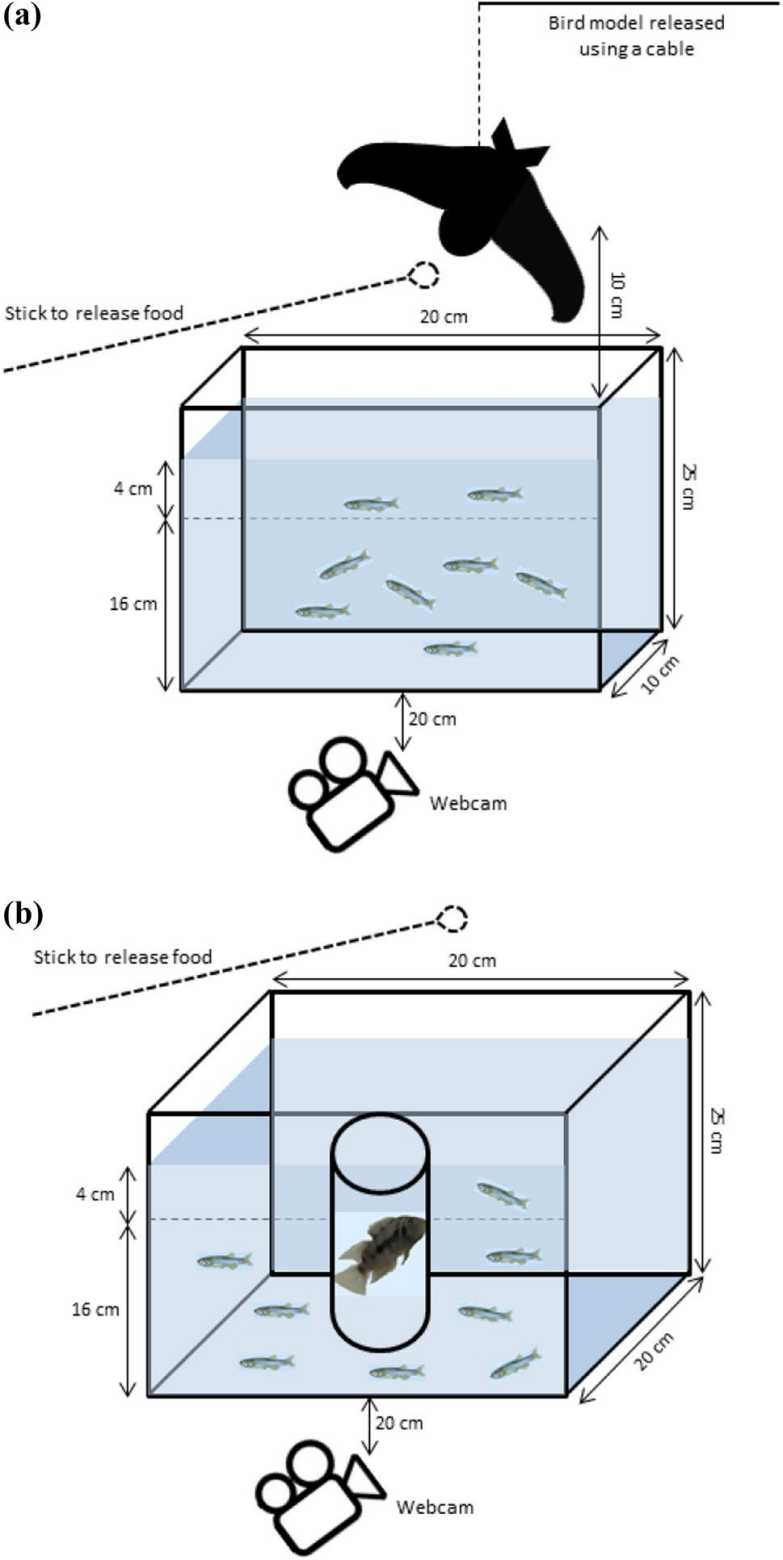
Experimental setups for testing risk-taking to feed in presence of **a** simulated aerial predator (in the form of a paper cutout of bird), and **b** a live convict cichlid fish. The image of zebrafish have been taken from Guerreiro (2008)

We used a rectangular glass tank (30 × 10 × 25 cm) as the experimental setup with white opaque walls on three sides and placed the setup behind a white curtain to avoid external disturbances affecting fish behaviour (Fig. 2a). The tank was filled with system water up to a level of 20 cm and we demarcated 4 cm from the top as the ‘surface zone’. We starved the fish before the experiments to equalize hunger levels so that they are motivated enough to feed. We gently transferred a group of eight fish from their holding into the arena and started recording their behaviour with a webcam (LogitechB910) installed 20 cm from the transparent side of the setup. After 2 min, we added food to the surface of water and allowed the fish to feed for 30 s. We released a paper cutout of a bird (simulated predator) at a height of 10 cm from the water surface using a cable cord so that it hovered over the tank i.e. remained in one place suspended by the cord in the air for 15 s (Fig. 2a). We then retrieved the model back and allowed 5 min for the fish to resume feeding. From the video recordings, we manually scored the cumulative time i.e. the total time spent by one or more fish at the surface (Egan et al. 2009; Kalue 2017) during the 5 min period while feeding after we retrieved the predator model as a measure of boldness.

### Risk-taking in presence of a live predator

We tested risk-taking to feed in presence of an aquatic predator, a convict cichlid (*Amatitlania nirgrofasciata*), twice (at 90–100 dpf and 132–142 dpf age) among adult zebrafish groups. Previous studies testing risk-taking behaviour in zebrafish have used convict cichlids (Sailer et al. 2012; Toms and Echevarria 2014). Though convict cichlid is not native to zebrafish habitats in India, other cichlids are found in zebrafish habitats (Engeszer et al. 2007). We used 42 groups (7 from each replicate line, 14 per treatment) or 336 fish in total. We used a similar setup like previous with a rectangular glass tank (30 × 20 × 25 cm) having three opaque walls (Fig. 2b) and a demarcated surface zone. We tested zebrafish in three different contexts like for other fish species (Mikheev et al. 2006) where we exposed them to only visual, only olfactory and synergistic (visual + olfactory) cues from the cichlid. For this, we introduced a cichlid fish into a cylindrical container that was transparent (for visual cue) and permeable (for both visual and chemical cues) or opaque and permeable (for chemical cue), and placed the container in the center of the experimental arena leaving it always in the same place (Fig. 2b). We also tested fish in a controlled setting without the predator. During the experiment, we first transferred the cichlid into the container and allowed it to acclimate for 30 min. We then transferred a group of eight fish into the arena and started recording their behaviour. After 2 min, we added food on the water surface and allowed the fish to feed for 5 min. From the video recordings, we manually scored the cumulative time spent by zebrafish at the surface.

### Statistical analysis

We constructed linear mixed-effects regression models (lmer) to test for difference in boldness among selection lines through ontogeny under aerial predation threat, and across different contexts in presence of live predator. In the test for risk-taking under simulated aerial predation, we transformed the response variable (cumulative time spent at the surface) using cube-root transformation and confirmed the normality and heterogeneity of the residuals. We then fitted mixed effects models using the transformed measure as a dependent variable, interaction of ‘Selection line’ (large-harvested, control and small-harvested) and ‘Age’ (ontogenetic stage) as the fixed effect and ‘Group ID’ nested within ‘Replicate’ (two per line) as random intercept (Formula: CuberootTime ~ Selection Line × Age + (1 | Replicate/Group ID)). To test for consistency in boldness across life-history stages, we estimated the adjusted repeatability (Nakagawa and Schielzeth 2010) for each selection line separately over three stages; larval (8–22 dpf), juvenile—subadult (46–85 dpf) and adult (105–190 dpf) using the ‘rpt’ function. We considered the cube-root transformed measure as dependent variable, ‘Age’ as fixed effect, and ‘Group ID’ as random effect. We used 95% confidence intervals with a significance level of 5% as estimates of uncertainty. To estimate variability and plasticity in boldness in each line, we used between-group and within-group variances (Polverino et al. 2016b; Roy et al. 2017) obtained by running separate mixed-effects regression models at each stage. We judged whether the variance estimates accounting for behavioural variability and plasticity differed considerably across selection lines and across ontogenetic stages by checking the non-overlap of confidence intervals.

To investigate differences in boldness across contexts in presence of a live predator, we first log(cumulative time + 1) transformed the response variable and confirmed the normality and heterogeneity of the residuals. We then fitted mixed effects models using the log(response + 1) transformed measure as the dependent variable, interaction of ‘Selection line’ and ‘Context’ (control, visual, chemical and visual + chemical) as the fixed effect, ‘Group ID’ nested within ‘Replicate’ as random intercept and ‘Age’ as the continuous random effect (Formula: LogTime ~ SelectionLine × Context + (Age | Replicate/Group ID)). In the event of a significant effect of selection line or context or their interaction, we conducted Tukey post hoc tests to evaluate which contexts differed among the selection lines. To test for consistency in boldness across four contexts, we estimated the adjusted repeatability (with 95% CI) like previous for each selection line by considering the log(response + 1) transformed measure as dependent variable, interaction of ‘Context’ and ‘Age’ as fixed effect, and ‘Group ID’ as random effect. To estimate behavioural variability and plasticity in each line, we similarly obtained between- and within-group variances by running separate mixed-effects regression models across contexts, and deduced whether these estimates differed across lines by checking the non-overlap of confidence intervals.

All analyses were conducted in R version 3.6.1 (R Development Core Team 2019). Mixed effects models were constructed using ‘*lmerTest*’ package (Kuznetsova et al. 2017), behavioural repeatability was estimated using ‘*rptR*’ package (Stoffel et al. 2017) and post hoc tests were conducted using ‘*emmeans*’ package (Lenth et al. 2018). Box-whisker plots were made using ‘*ggplot2*’ (Wickham 2011) and ‘*ggpubr*’ (Kassambara and Kassambara 2020) packages in R.

## Results

**Fig. 3:**
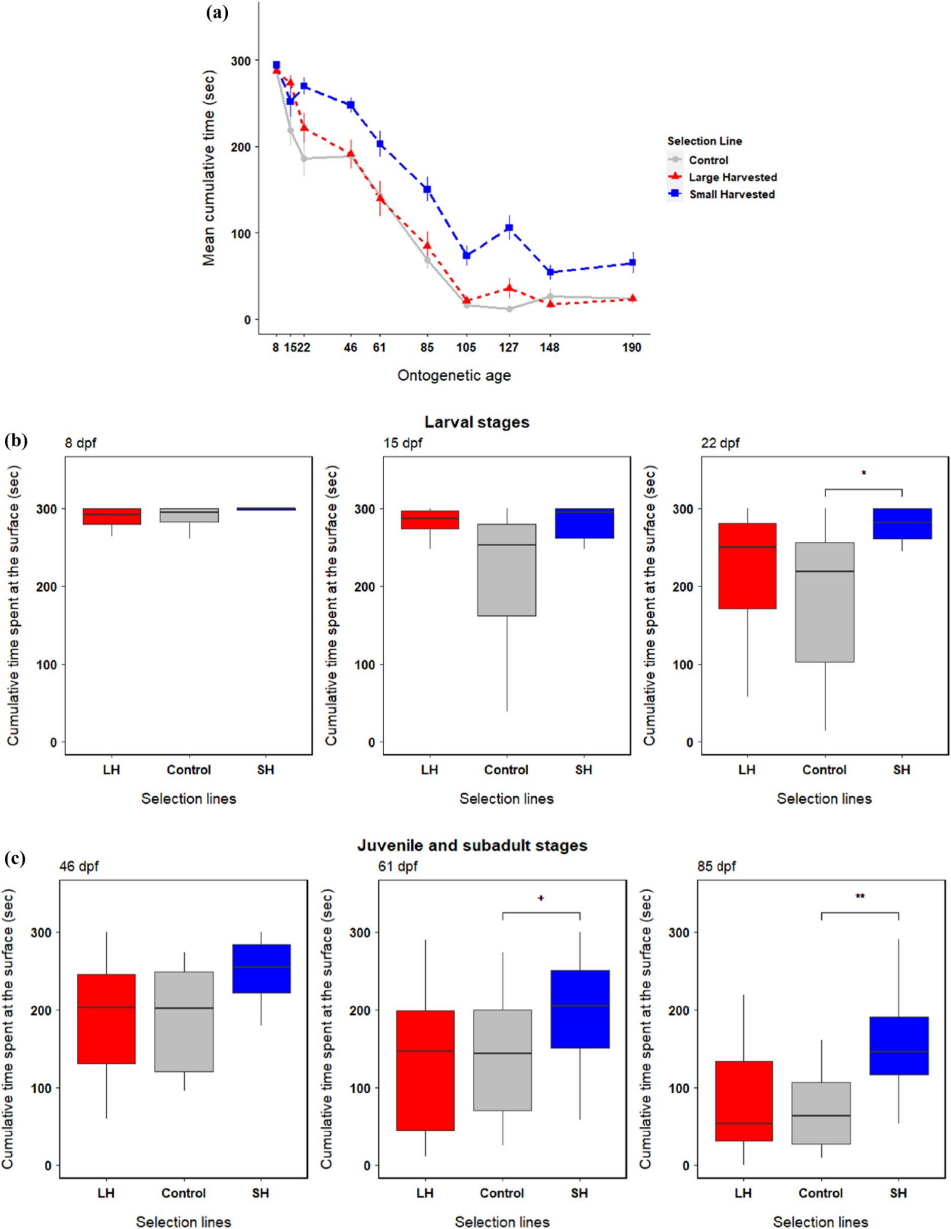

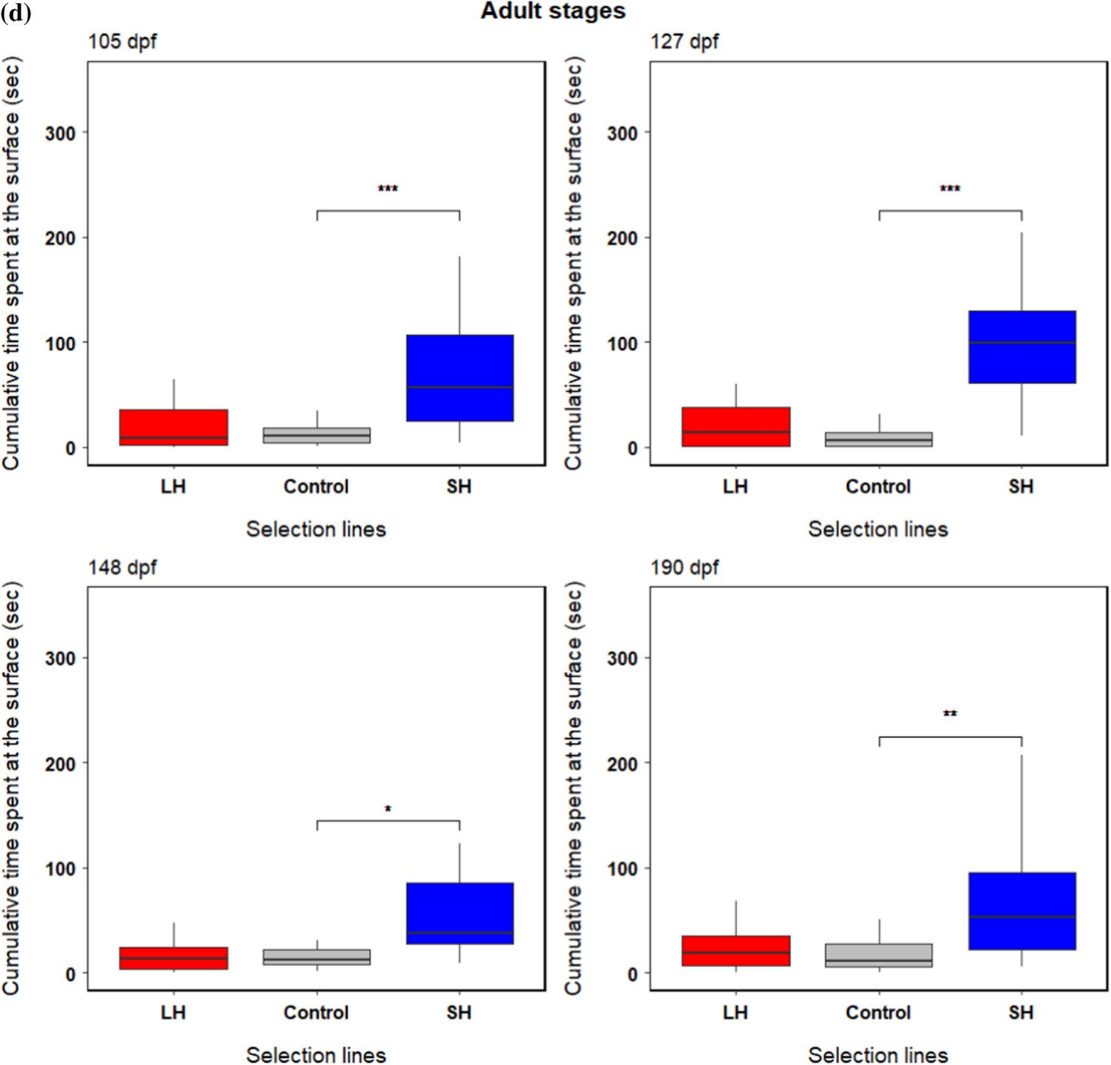
Change in boldness (measured as cumulative time spent at the surface) through ontogeny among large-harvested (LH: red), control (grey) and small-harvested (SH: blue) selection lines (*N* = 60 groups). The first panel **a** shows change in mean cumulative time (± SE) spent at the surface across all ontogenetic stages (8 to 190 dpf). The second panel **b** shows behavioural change across larval stages from 8 to 22 dpf. The third panel **c** shows behavioural change across juvenile (~ 46 dpf when metamorphosis is complete) and subadult stages at 61 and 85 dpf. The fourth panel **d** shows behavioural change across adult stages from 105 to 190 dpf. Significant differences are indicated with bars and codes ***(*p* < 0.001), **(*p* < 0.01), *(*p* < 0.05), and ^**+**^(*p* < 0.1)

**Fig. 4:**
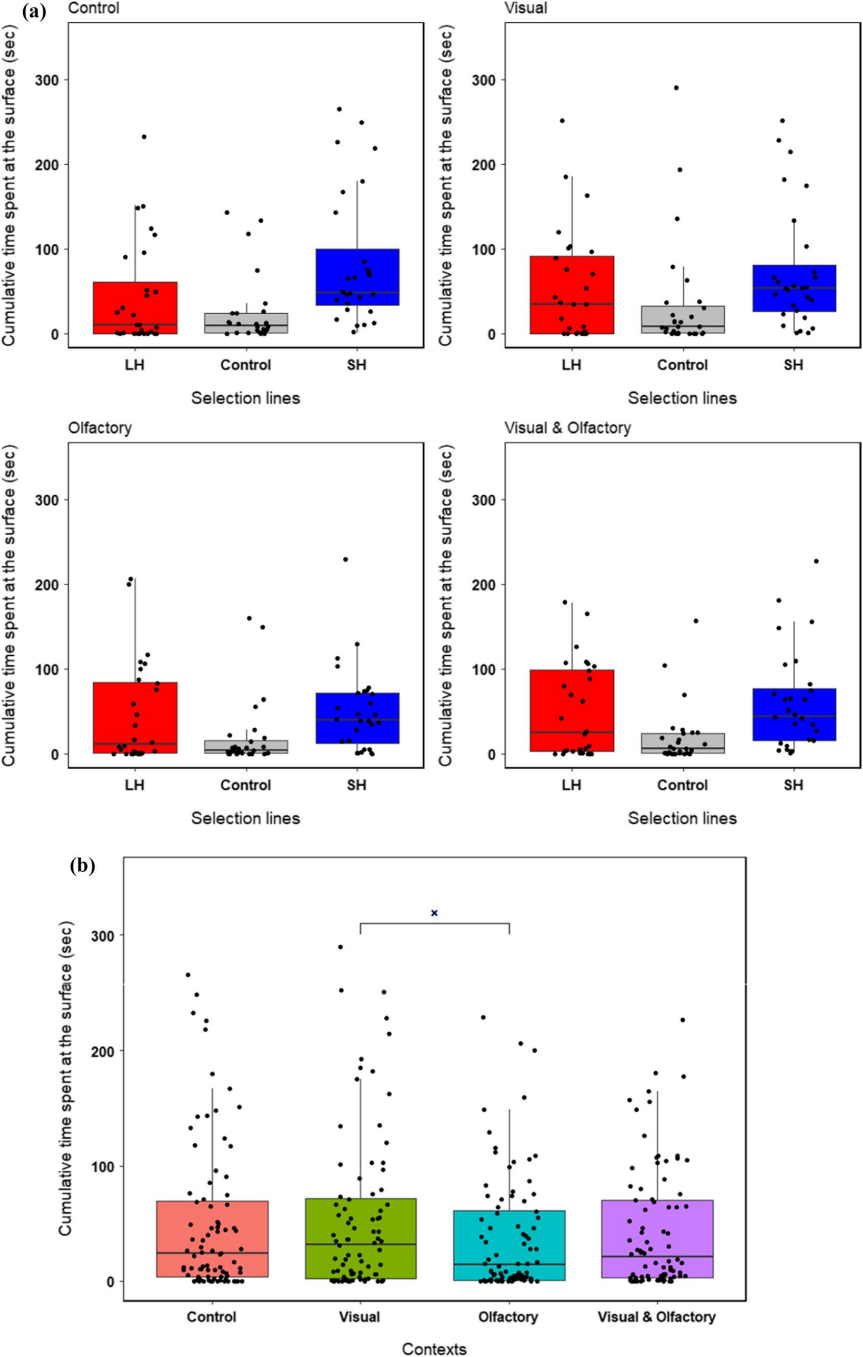
Combined plot (for 90–100 dpf and 132–142 dpf age) of cumulative time spent at the surface (boldness) by **a** zebrafish across selection lines and **b** all fish across three contexts where they perceived visual, olfactory and synergistic (visual + olfactory) cues from the cichlid fish, and in a control setting without the predator (*N* = 42 groups). A significant difference is indicated with a bar and code × (*p* = 0.05)

We found a significant and line-specific ontogenetic effect on group boldness (selection line × age; *F*_18, 513_ = 5.32, *p* < 0.01) (Table 1a) meaning that group boldness (risk-taking behaviour) changed significantly through ontogeny across selection lines (Fig. 3a). The cumulative time spent by fish at the surface decreased significantly as the fish matured and developed through ontogeny, i.e., bigger and older fish generally took less risk to feed than smaller and younger individuals (Fig. 3a–d, 4). The small-harvested line fish were significantly bolder (i.e. spent more time at the surface feeding at the surface) than the control (random-harvested) line fish at larval stage 22 dpf (*t* = 2.21, *p* = 0.03) (Fig. 3b), subadult stages 61 dpf (*t* = 1.77, *p* = 0.08) and 85 dpf (*t* = 3.05, *p* < 0.01) (Fig. 3c), and adults stages 105 dpf (*t* = 4.08, *p* < 0.01), 127 dpf (*t* = 6.82, *p* < 0.01), 148 dpf (*t* = 2.47, *p* = 0.01) and 190 dpf (*t* = 3.06, *p* < 0.01) (Fig. 3d). The large-harvested line fish did not differ in boldness from the control line fish at any developmental stage (Fig. 3a–d, Table 1b). Hence, while all lines decreased their boldness levels as the fish aged (Fig. 3a), the decrease in boldness after maturation was less pronounced in the small-harvested line (Fig. 3a, d). Boldness levels decreased more in the control and large-harvested line fish and both lines became particularly shy after maturation compared to the juvenile and larval stages (Fig. 3a–d).

**Table 1:** Results of (a) main effects and (b) fixed effects terms obtained from linear mixed effects model comparing boldness in fish from selection lines LH (large-harvested) and SH (small-harvested) with the control (random-harvested) line across ontogenic stages A (8 dpf) to J (190 dpf). Significance of the fixed effects and their interactions are in bold (marginal: ‘^+^’)

(a) Main effects	Sum Sq	Mean Sq	NumDF	DenDF	*F* value	Pr(> *F*)
Selection line	7.56	3.780	2	3.00	4.65	0.12
Age	1399.40	155.49	9	513.16	191.42	** < 0.001**
Selection line × age	77.73	4.32	18	513.16	5.317	** < 0.001**

(b) Fixed effects	Estimate	Std. Error	df	*t* value	Pr( >|*t*|)
Intercept	6.60	0.34	6.55	19.63	** < 0.001**
Selection line: LH	− 0.004	0.47	6.55	− 0.01	0.99
Selection line: SH	0.05	0.47	6.55	0.11	0.91
Stage B (15 dpf)	− 0.71	0.28	512.34	− 2.49	**0.01**
Stage C (22 dpf)	− 1.10	0.28	512.34	− 3.88	** < 0.001**
Stage D (46 dpf)	− 0.95	0.29	518.59	− 3.32	** < 0.001**
Stage E (61 dpf)	− 1.57	0.28	512.34	− 5.50	** < 0.001**
Stage F (85 dpf)	− 2.76	0.28	512.34	− 9.70	** < 0.001**
Stage G (105 dpf)	− 4.39	0.28	512.34	− 15.40	** < 0.001**
Stage H (127 dpf)	− 4.90	0.28	512.34	− 17.20	** < 0.001**
Stage I (148 dpf)	− 4.06	0.28	512.34	− 14.26	** < 0.001**
Stage J (190 dpf)	− 4.14	0.28	512.34	− 14.51	** < 0.001**
LH × Stage B	0.59	0.40	512.34	1.46	0.14
SH × Stage B	0.27	0.40	512.34	0.66	0.51
LH × Stage C	0.45	0.40	512.34	1.11	0.27
SH × Stage C	0.89	0.40	512.34	2.21	**0.03**
LH × Stage D	− 0.02	0.40	512.34	-0.05	0.96
SH × Stage D	0.56	0.40	512.34	1.40	0.16
LH × Stage E	− 0.15	0.40	512.34	-0.38	0.71
SH × Stage E	0.71	0.40	512.34	1.77	0.08^+^
LH × Stage F	0.10	0.40	512.34	0.24	0.81
SH × Stage F	1.23	0.40	512.34	3.05	** < 0.01**
LH × Stage G	− 0.15	0.40	512.34	-0.36	0.71
SH × Stage G	1.65	0.40	512.34	4.08	** < 0.001**
LH × Stage H	0.65	0.40	512.34	1.62	0.10
SH × Stage H	2.75	0.40	512.34	6.82	** < 0.001**
LH × Stage I	− 0.36	0.40	512.34	-0.91	0.36
SH × Stage I	0.99	0.40	512.34	2.47	**0.01**
LH × Stage J	0.00	0.40	512.34	0.01	0.99
SH × Stage J	1.23	0.40	512.34	3.06	** < 0.01**

When examining the behavioural consistency across life-history stages, the selection lines showed no significant repeatability in group boldness across larval stages i.e. from 8 to 22 dpf (Table 2a). Both large-harvested and small-harvested lines showed higher within-group variances i.e. higher behavioural plasticity compared to the control line (Table 2a). From juvenile (46 dpf) to subadult (85 dpf) stages, significant repeatability was observed in the large-harvested (*R* = 0.48, *p* < 0.001) and small-harvested (*R* = 0.43, *p* < 0.001) lines while the control line fish showed no significant repeatability in group boldness (Table 2b). The small-harvested line exhibited lower within-group variance, i.e. lower behavioural plasticity compared to the control line (Table 2b). In adults from 105 to 190 dpf, significant repeatability in boldness was observed in the large-harvested (*R* = 0.61, *p* < 0.001), control (*R* = 0.48, *p* < 0.001) and small-harvested (*R* = 0.57, *p* < 0.001) lines (Table 2c). Compared to the larval stages, we found higher behavioural variability in all stages in the large-harvested line, and in the adult stages in control and small-harvested lines (Table 2). Behavioural plasticity was higher in the large-harvested line in all stages compared to the larval stages (Table 2).

**Table 2:** Between- and within-group variances and repeatability in boldness among selection lines across (a) larval (8 to 22 dpf), (b) juvenile and subadult (46 to 85 dpf), and (c) adult stages (105 to 190 dpf)

Selection line	Between-group variance (CI)	Within-group variance (CI)	*R* (CI)	SE	*p*
(a) Larval stages					
Large-harvested	0.05 (0, 0.41)	0.24 (0.39, 0.61)	0.16 (0, 0.45)	0.13	0.12
Control	4.22e^−16^ (0, 0.48)	7.64e^−1^ (0.72, 1.03)	0.00 (0, 0)	0.00	0.5
Small-harvested	0.00 (0, 0.32)	0.31 (0.46, 0.66)	0.00 (0, 0.28)	0.08	1
(b) Juvenile and sub-adult stages					
Large-harvested	0.77 (0.51, 1.32)	0.83 (0.72, 1.12)	0.48 (0.19, 0.71)	0.14	** < 0.001**
Control	0.00 (0, 0.54)	0.90 (0.78, 1.12)	< 0.01 (0, 0.28)	0.08	1
Small-harvested	0.28 (0.30, 0.81)	0.37 (0.48, 0.75)	0.43 (0.13, 0.66)	0.14	** < 0.001**
(c) Adult stages					
Large-harvested	1.19 (0.75, 1.56)	0.76 (0.72, 1.03)	0.61 (0.37, 0.78)	0.11	** < 0.001**
Control	0.55 (0.48, 1.09)	0.59 (0.63, 0.91)	0.48 (0.24, 0.68)	0.12	** < 0.001**
Small-harvested	0.62 (0.53, 1.14)	0.47 (0.56, 0.81)	0.57 (0.35, 0.75)	0.10	** < 0.001**

The variances and repeatability (*R*) estimates are shown with 95% CIs in parentheses, and the SE and *p* values correspond with the repeatability estimates. Significant results are in bold

In the test for boldness in presence of a live predatory fish, we found a significant effect of context (*F*_3,243_ = 2.56, *p* = 0.05) but no significant effect of selection line (*F*_2,3_ = 1.58, *p* = 0.34) on collective boldness (Table 3a, Fig. 4a, b). Post-hoc tests comparing boldness across contexts revealed that time spent feeding in all contexts when the predator was present did not differ significantly from the control (Table 3b, Fig. 4b). However, zebrafish spent more time feeding when they perceived only visual rather than olfactory cues from the cichlid (Table 3b, Fig. 4b). In tests for behavioural consistency, we found significant and very high repeatability in boldness across four contexts and across age among large-harvested (*R* = 0.87, *p* < 0.001), control (*R* = 0.53, *p* < 0.001) and small-harvested (*R* = 0.54, *p* < 0.001) line fish (Table 4). Large-harvested line fish showed lower within-group variance, i.e. lower behavioural plasticity across contexts and time compared to the control line (Table 4).

**Table 3:** Results of (a) main effects from the linear mixed effects model comparing boldness in presence of live predator in fish from three selection lines across four contexts (‘Control’, ‘Visual’, ‘Olfactory’ and ‘Visual & Olfactory’), and (b) post hoc tests comparing cumulative time across four contexts averaged over the level of selection lines

(a) Main effects	Sum Sq	Mean Sq	NumDF	DenDF	*F* value	Pr(> *F*)
Selection line	2.66	1.33	2	3	1.58	0.34
Context	6.49	2.16	3	243	2.56	**0.05**
Selection line × Context	7.89	1.31	6	243	1.56	0.16

(b) Pairwise differences of contexts	Estimate	Std. Error	df	*t* ratio	*P* value
Control—Visual	− 0.05	0.14	243	− 0.35	0.98
Control—Olfactory	0.31	0.14	243	2.21	0.12
Control—Visual & Olfactory	0.09	0.14	243	0.61	0.93
Visual—Olfactory	0.36	0.14	243	2.56	**0.05**
Visual—Visual & Olfactory	0.14	0.14	243	0.96	0.77
Olfactory—Visual & Olfactory	− 0.23	0.14	243	− 1.59	0.38

Significant results are in bold

**Table 4:** Between- and within-group variances and repeatability in boldness across contexts among selection lines

Selection line	Between-group variance (CI)	Within-group variance (CI)	*R* (CI)	SE	*p*
Large-harvested	3.61 (1.30, 2.81)	0.55 (0.63, 0.83)	0.87 (0.72, 0.94)	0.05	** < 0.001**
Control	1.40 (0.77, 1.80)	1.23 (0.95, 1.25)	0.53 (0.27, 0.73)	0.12	** < 0.001**
Small-harvested	0.95 (0.64, 1.48)	0.80 (0.75, 1.00)	0.54 (0.29, 0.73)	0.12	** < 0.001**

The repeatability (*R*) and variance estimates are shown with 95% CIs, and the SE and *p* values correspond with the repeatability estimates. Significant results are in bold

## Discussion

Our study revealed that five generations of size-selective harvesting followed by 10 generations of no selection left an evolutionary legacy on collective risk-taking behaviour (boldness) in zebrafish at different levels of predation risk. Under aerial predation threat, boldness levels decreased with ontogenetic age and fish became shyer after maturation compared to the juvenile and larval stages. This effect was least pronounced in the small-harvested line as these fish were consistently bolder than the controls post the larval stages (8–22 dpf). Large- and small-harvested lines showed consistency in boldness beyond juvenile and subadult stages (46–85 dpf) but all lines showed consistency in boldness in the adult stages (105–190 dpf). Thus, both positive and negative size-selection fostered early emergence of collective personality with respect to the control line. Positive and negative size-selection favoured higher behavioural plasticity in the larval stages, and positive size-selection led to increased variability and plasticity in boldness in the juvenile and adult stages compared to the larval stages. The line differences in boldness in presence of an aerial predator were, however, eliminated in presence of a predatory fish. Here, fish across all lines showed consistency, and the large-harvested line fish showed lower plasticity in boldness across contexts. The genetic, morphological and physiological differences that the selection lines harboured even after selection was stopped (Roy et al. 2021; Sbragaglia et al. 2021; Uusi‐Heikkilä et al. 2017) could be the major contributor to differences in personality expression, and variability and plasticity.

We found that boldness levels decreased significantly with increase in ontogenetic age across all selection lines. This meant that as fish matured from larval to the adult stages, they took significantly less risk to feed on the water surface following the unprecedented disturbance overhead due to sudden release and retrieval of the predator model. We expected this pattern only in the large-harvested line where we predicted that fish would become shyer as adults to adjust their behaviour to the smaller body-size but we found this across all lines. The reason for decline in boldness with maturity is potentially based on the asset protection hypothesis according to which adults will be less prone to take risks for safeguarding possibilities for future mating while larval fish will take more risks because they have least investment in reproductive assets like gonads (Wolf et al. 2007). There could be other reasons behind these results. First, the motivation for feeding increases in larval zebrafish after the absorption of yolk resulting in larvae becoming more voracious feeders. Adults with greater energy reserves and gut capacity may not require foraging as intensively as larval or juvenile fish (Fuiman and Webb 1988). Also, larval and juvenile fish have greater metabolic rates, lower body fat reserves and higher drag coefficients and therefore may be more inclined to take risks to feed than adults (Krause et al. 1998; Wootton 1994). Secondly, larval fish may perceive a looming stimulus like a predator model approaching overhead differently than adults (Fero et al. 2011). This could be possibly due to underdeveloped sensory and motor systems compared to adults (Fuiman and Magurran 1994). Larval fish showed a startle response after the simulated attack but were quicker to swim back to the surface unlike adults. Adults with developed visual and sensory systems are more vigilant, have a better knowledge of the risk-zone (surface) and may swim up to the surface only when hungry. Thirdly, larval stages may not be the target of large predators like birds and it is only when fish are adults that they face avian predation (Fuiman and Magurran 1994). This could also be a reason why the larval fish spent more time at the surface than the adults. Fourth, adult zebrafish show more shoaling behaviour than larval or juvenile zebrafish (Fuiman and Webb 1988; Miller and Gerlai 2011). Because we tested fish in groups, larval and juvenile fish due to their low shoaling tendency had higher probabilities of leaving the association of groups for foraging. Contrarily, adults due to their increased shoaling tendency might have been more reluctant to leave the association of group for foraging. Our results contradict previous studies on mosquitofish that showed that juveniles showed decreased boldness compared to adults (Polverino et al. 2016b, 2016c), and partially agree with a study on mangrove killifish (*Kryptolebias marmoratus*) where fish became bolder during early development followed by a reduction in boldness post sexual maturity (Edenbrow and Croft 2011). The reasons for this difference could be that these studies tested latency of individuals to emerge out of a shelter in an open field and did not measure boldness in the context of foraging in groups. Species-specific differences might also be responsible for the observed variations.

We found that beyond the larval stages (i.e., 46 dpf onward), the small-harvested line fish were significantly bolder than the control line fish while the large-harvested line fish did not differ in group boldness compared to the controls. This supported our hypothesis and the theoretical model by Andersen et al. (2018) that negative size-selection will lead to elevated boldness in zebrafish. The fact that the large-harvested line fish did not differ in boldness but the small-harvested line showed elevated boldness relative to the control line is in partial agreement with Andersen et al. (2018) which predicted that unless very large-sized fish are harvested, any kind of selective harvesting would foster boldness. Our results on juveniles (46 dpf) are supported by previous findings of Uusi‐Heikkilä et al. (2015) where juvenile individuals (30 day old) of the small-harvested, and not the large-harvested line were bolder than the control line, though the study implemented an open-field assay to test risk-taking behaviour in individuals and not groups. Higher risk-taking to feed in juveniles in the small-harvested line could be justified by the need to develop energy reserves for investment in growth following the energy acquisition pathway (Enberg et al. 2012). On the other hand, no difference in boldness in the large-harvested compared to controls would mean that though this line evolved a fast life-history (Uusi‐Heikkilä et al. 2015), this does not necessary lead to higher foraging tendency to build energy reserves necessary for early gonadal investment. Further, our results with sub-adults (61–85 dpf) and adults (105–190 dpf) showing increased boldness in the small-harvested line again only partly agrees with our expectations that both lines would show elevated boldness in response to size-selection (Andersen et al. 2018). These results are in agreement with a previous study by Sbragaglia et al. (2021) which implemented a similar assay to test group risk-taking among zebrafish selection lines and found that boldness was higher among adults of the small-harvested line while the large-harvested line fish did not consistently differ in boldness compared to the control line fish. Our results are however in contrast with the findings of Sbragaglia et al. (2019a) where individual adult females of the small-harvested line showed lower risk-taking tendency in an open-field test compared to the control line fish. An open field test measures exploratory behaviour rather than boldness (Réale et al. 2010) and the assay does not consider the vertical dimension of fish movement. Considering this dimension is important because zebrafish in holding are fed at the surface. The results with subadults and adults, like juveniles in the small-harvested line fish, could be reasoned based on energy acquisition mechanism and the fast growing fish of the small-harvested line may need to forage more to achieve their endpoints (Enberg et al. 2012). Moreover, body-size in adult zebrafish is positively associated with boldness (Polverino et al. 2016a; Roy and Bhat 2018a) perhaps due to lower predation risk in larger fish, and the small-harvested line fish being larger in size compared to the other two lines are therefore bolder as adults.

Within the large-harvested line, we expected that fish would become relatively shyer as adults compared to the control line to adjust their behaviour to their relatively smaller adult body-size. Although we saw a change in boldness from larvae to adults, this was not significantly different from the control line (Fig. 4a). Thus, our work did not support the expectations of the timidity-syndrome hypothesis that size-selection alone leads to shyness (Arlinghaus et al. 2017). The fact that we did not see behavioural adaptations in the large-harvested line compared to the controls despite a strong life-history adaption (Uusi‐Heikkilä et al. 2015) may be because of evolutionary resistance to change in behaviour. This means that boldness levels in fish may not necessarily drop below certain levels as a result of strong size-selection even though a significant life-history change was observed. A similar asymmetrical selection response was demonstrated in a study on medaka (*Oryzias latipes*) where positive (fishing like) selection of size did not have an effect on the life-history traits but a negative selection of size and maturity had a strong impact on life-history (Renneville et al. 2018). Similarly, the large-harvested line in our study did not show reduced juvenile growth rate compared to controls (Uusi‐Heikkilä et al. 2015) which is a typically expected evolutionary outcome of size-selective mortality (Conover and Munch 2002). Thus, selection on one trait may not be associated with symmetrical changes in other functional traits (Bartuseviciute et al. 2022). We propose that for group shyness to evolve in response to fishing, selection must operate directly on that behaviour. Another explanation could be that as the lines were no longer under selection since 10 generations, this might have caused some trait recovery in them (Conover et al. 2009; Salinas et al. 2012). A previous study on silverside fish *Menidia menidia* showed that populations that evolved smaller body-size after five generations of large-size harvesting showed a steady trait reversal after the size-selection was stopped (Conover et al. 2009). Our results of no change in boldness in the large-harvested line compared to the controls 10 generations after selection was stopped could be reasoned out similarly.

We found no consistency in collective boldness in the larval stages (8–22 dpf) in any of the selection lines while high consistency in boldness in adults (105–190 dpf) among all lines. These results indicate emergence of collective personality with ontogeny in zebrafish, similar to reports from other fish species (Edenbrow and Croft 2013; Polverino et al. 2016b), and development of shoaling behaviour with ontogenetic age in zebrafish (Buske and Gerlai 2011; Mahabir et al. 2013). In larval stages, zebrafish do not rely on social information and have higher tendency to move away from groups and find resources on their own (Fuiman and Webb 1988). This pattern changes with maturation when zebrafish develop attraction strength and start shoaling (Hinz and de Polavieja 2017) that results in more consistent behaviour. Importantly, we found early emergence (from the juvenile stage onwards) of collective personality in the large- and small-harvested lines. Because repeatability is the proportion of behavioural variation attributable to interindividual/intergroup differences (Dingemanse and Dochtermann 2013), existence of behavioural variation in fish groups of large and small-harvested lines in these ontogenetic stages (absent in the control line, Table 2b) could have manifested in the early development of personality. We further found higher plasticity in boldness during larval stages (8–22 dpf) in the large- and small-harvested lines, but lower plasticity from juvenile to subadult (46–85 dpf) stages in the small-harvested line, compared to the controls. This meant that both positive and negative size-selection caused behavioural plasticity to set in very early in life. Individual-level differences translate to within-group variation in behaviour (Jolles et al. 2017), previously reported in the zebrafish lines for expression of size-at-age variation in response to harvest-induced selection (Uusi-Heikkilä et al. 2016). The elevated plasticity in boldness of individual larvae could have contributed to the high plasticity in fish groups of large- and small-harvested lines that we report. The reasons behind plasticity in the larval stages in the large and small-harvested lines could be differences in internal states leading to different tendencies to forage (Dingemanse and Wolf 2013), and maintenance and fostering of multiple pathways (physiological or behavioural) to reach same fitness goals under strong selection (Kobler et al. 2009). Differences in internal states and expression of physio-behavioural types were perhaps counterbalanced in the control line fish due to the random nature of size selection, while the fish in the large and small-harvested lines might have been forced to develop variable responses to reach the same fitness outcome leading to plasticity in turn. As the control line fish also showed increased plasticity in boldness beyond the larval stages, the relative difference in within-group variances among the selection lines decreased and the small-harvested line was found to be less plastic.

The large-harvested line fish showed higher variability and plasticity in boldness from juvenile to adult stages compared to the larval stages. This is similar to studies where fisheries-induced selection in pike (Edeline et al. 2009), and positive size-selective mortality in zebrafish (Uusi-Heikkilä et al. 2016), led to higher variability in morphological traits (size-at-age). The reason behind increased behavioural variability and plasticity through development could be increased differences in the internal state and behaviour of individuals comprising the groups. These differences among individuals could be because of the internal conflict between reaping resources through foraging for allocation into reproduction and avoiding being predated upon due to the smaller body size. Recent models by Sbragaglia et al. (2022) showed increased vigilance among adults of the large-harvested line and this could also be responsible for the interindividual differences. Theoretical models (Fawcett and Frankenhuis 2015; Fischer et al. 2014) and studies in other fish species (Bierbach et al. 2017; Polverino et al. 2016b) showed a decrease in behavioural plasticity through ontogeny. Our result shows an opposite trend which emphasizes the impact of positive size-selection on behavioural plasticity. Behavioural variability was generally higher in adults of all selection lines compared to the larval stages meaning that as zebrafish matured, individuals in groups became increasingly divergent in their behaviour. Aerial predators are perceived as a threat by adults rather than larval fish due to less developed sensory abilities (Fuiman and Magurran 1994). Hence, the larval fish perhaps needed to be less variable in risk-taking tendencies than adults that were more frightened by the simulated predator leading to higher variation among groups.

In presence of a live predator, fish did not differ in boldness relative to control in each of the three contexts meaning that group boldness was unaffected by presence of convict cichlid as a predatory fish. Antipredator responses in fish to different cues from predators are driven by early experiences (Jonsson and Jonsson 2014). As our fish are laboratory reared and have not experienced aquatic predators before, the different cues from the predator did not have a variable impact on them. Zebrafish were bolder when perceiving visual than olfactory cues but these results seem to be less important as the responses do not differ from control. Importantly, the observed differences in group boldness between adults of the small-harvested and control lines under aerial predation threat disappeared here. This reinforces previous findings in fish that aerial and aquatic predators exert different selection pressures (Godin and Clark 1997; Templeton and Shriner 2004) and thus induces different behavioural responses in prey. Birds attack the water surface (Doran et al. 2022) while predatory fish may dive all along the water-column to catch their prey and this may cause prey fish to adopt different antipredator strategies (Fuiman and Magurran 1994; Templeton and Shriner 2004). Our work raises caution that paying attention to different predation strategies of different predators is critical when testing boldness in fish.

All selection lines showed consistent differences in collective personality across contexts and time, like adults under simulated aerial predation threat. This is similar to previous studies in wild zebrafish showing consistent differences in individual personality across contexts that differed in predation threat (Roy et al. 2017), and time (Roy and Bhat 2018b). Consistent differences in internal states of individuals due to uniform hunger levels, familiarity among individuals leading to repeated social interactions, and similar non-familiarity among all fish with the predator could be the reasons why the groups varied consistently across contexts in our study. The large-harvested line showed lower behavioural plasticity than the control line (Table 4) meaning that the within-group variation in risk-taking tendencies was similar across contexts and age. Low plasticity may provide less fitness advantages in response to changing predation threats (Dingemanse and Wolf 2013) and can have detrimental consequences for population survival. A model by Sbragaglia et al. (2022) suggested that the natural mortality of the large-harvested line might be elevated. Further studies exposing the selection lines to direct predation are warranted to investigate if they vary in survival against natural predators.

Although we see differences in collective boldness and its variability and plasticity among selection lines in two different experiments, we cannot ignore the fact that as the lines were no longer under selection since 10 generations, this might have caused some trait recovery in them. Previous studies on Atlantic silverside have shown that cessation of selection for a couple of generations after size-selective harvesting caused evolutionary changes in life-history traits led to partial to complete trait recovery (Conover et al. 2009; Salinas et al. 2012). But food consumption rate in silversides did not return to pre-harvesting levels after selection was stopped indicating that this trait could have been evolutionary fixed due to size-selection (Salinas et al. 2012). This offers support for our results and indicates that stopping selection might not have hampered feeding behaviour. That said, it is possible that the observed variations among selection lines are due to other factors like density (Bouffet‐Halle et al. 2021; Crespel et al. 2021a, b) and genetic drift (Therkildsen et al. 2019). A recent study in medaka showed that life-history divergence may not be caused by direct harvest selection but by natural density-dependent selection for a larger body-size (Bouffet‐Halle et al. 2021). Though we maintained a uniform density of eight fish per box right from the embryonic stage and throughout the period of experiments, there could have been variations in fish density in the holding tanks during rearing and development. These differences in population developmental density may determine the evolutionary potential of size-selection (Crespel et al. 2021a). Further, genetic drift may cause differences between the selection line replicates (Therkildsen et al. 2019) and this may account for the variations among selection lines.

## Conclusions

Our results demonstrated that intensive size-selection for five generations followed by 10 generations of relaxed selection caused evolutionary fixed responses in terms of substantial changes in boldness as a collective personality trait. Collective personality emerged earlier in ontogeny as a result of positive and negative size-selection in zebrafish. Negative size-selective mortality fostered increased boldness compared to other forms of selection under aerial predation threat, and this might lead to increased natural mortality and be detrimental for populations, as predicted theoretically by Jørgensen and Holt (2013) and shown in a meta-analysis (Moiron et al. 2020). By contrast, no significant changes in group boldness were found in the large-harvested line, but other work has shown that group cohesion is reduced which might also lead to elevated natural mortality in the presence of predators (Sbragaglia et al. 2022). Positive size-selection representing harvesting patterns commonly observed in most commercial and recreational fisheries resulted in an increase in behavioural variability and plasticity across ontogeny, and reduced plasticity in presence of aquatic predation threat. These findings might be interpreted in the light of changes in vigilance through large-harvest selection (Sbragaglia et al. 2022), but it is an open question whether these affect relevant fitness outcomes such as natural mortality. Generally, our study provides empirical evidence for theoretical studies that predicted that evolution of boldness is conceivable due to size-selective harvesting alone (Andersen et al. 2018; Claireaux et al. 2018). Future studies testing cognitive learning and decision-making, and mortality in the face of real predation are required to understand the adaptive significance of the altered group risk-taking behaviour that we documented in our selection lines.

## Supplementary Information

Below is the link to the electronic supplementary material.

Supplementary file1 (DOCX 19 KB)

Supplementary file2 (XLSX 62 KB)

## Data Availability

The data collected and used in this study are available as supplementary material.
