# Supplementary material for: Size-selective mortality fosters ontogenetic changes in collective risk-taking behaviour in zebrafish, Danio rerio
Source: Oecologia. 2022 Oct 1;200(1-2):89–106. doi: 10.1007/s00442-022-05256-y (PMC9547785; doi:10.1007/s00442-022-05256-y)
Supplement: Supplementary file 1 — Supplementary file1 (DOCX 19 KB) [file 442_2022_5256_MOESM1_ESM.docx]

**Size-selective mortality fosters ontogenetic changes in collective risk-taking behaviour in zebrafish, *Danio rerio***

Tamal Roy^1^* and Robert Arlinghaus^1,2^

^1^Department of Fish Biology, Fisheries and Aquaculture, Leibniz Institute of Freshwater Ecology and Inland Fisheries, Müggelseedamm 310, 12587 Berlin, Germany

^2^Division of Integrative Fisheries Management, Department of Crop and Animal Sciences, Faculty of Life Sciences, Humboldt University of Berlin, Unter den Linden 6, 10099 Berlin, Germany

^*^Correspondence author; email: Tamal.Roy@igb-berlin.de


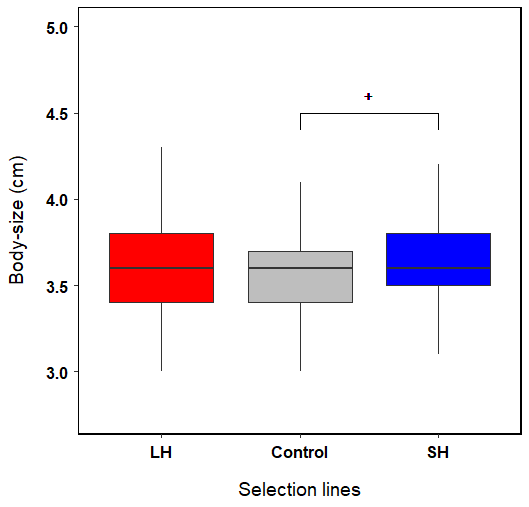


Figure 1: Comparison of body-size of non-experimental fish (11-month-old) across selection lines in F_16_ using 60 fish per replicate line (N=360 fish in total). We used linear models to compare body-size among selection lines. The small-harvested line (SH) fish were larger than the control (RH) line fish. Significant difference is indicated with code **^+^** (p=0.08).
